# Psychological effects of implantable cardioverter defibrillator shocks. A review of study methods

**DOI:** 10.3389/fpsyg.2015.00039

**Published:** 2015-02-04

**Authors:** Gian Mauro Manzoni, Gianluca Castelnuovo, Angelo Compare, Francesco Pagnini, Vidal Essebag, Riccardo Proietti

**Affiliations:** ^1^Psychology Research Laboratory, Istituto Auxologico Italiano IRCCSVerbania, Italy; ^2^Department of Psychology, Catholic University of MilanMilano, Italy; ^3^Department of Psychology, University of BergamoBergamo, Italy; ^4^McGill University Health CenterMontreal, QC, Canada; ^5^Cardiology Department, Luigi Sacco HospitalMilano, Italy

**Keywords:** implantable cardioverter defibrillator, ICD shock, quality of life, anxiety, depression, review

## Abstract

**Background:** The implantable cardioverter defibrillator (ICD) saves lives but clinical experience suggests that it may have detrimental effects on mental health. The ICD shock has been largely blamed as the main offender but empirical evidence is not consistent, perhaps because of methodological differences across studies.

**Objective:** To appraise methodologies of studies that assessed the psychological effects of ICD shock and explore associations between methods and results.

**Data Sources:** A comprehensive search of English articles that were published between 1980 and 30 June 2013 was applied to the following electronic databases: PubMed, EMBASE, NHS HTA database, PsycINFO, Sciencedirect and CINAHL.

**Review Methods:** Only studies testing the effects of ICD shock on psychological and quality of life outcomes were included. Data were extracted according to a PICOS pre-defined sheet including methods and study quality indicators.

**Results:** Fifty-four observational studies and six randomized controlled trials met the inclusion criteria. Multiple differences in methods that were used to test the psychological effects of ICD shock were found across them. No significant association with results was observed.

**Conclusions:** Methodological heterogeneity of study methods is too wide and limits any quantitative attempt to account for the mixed findings. Well-built and standardized research is urgently needed.

## Introduction

The implantable cardioverter defibrillator (ICD) has become the treatment of choice for both primary and secondary prevention of sudden cardiac death (SCD) due to ventricular arrhythmias (VA). Major clinical trials have consistently shown the ICD to be superior to antiarrhythmic drugs in patients at high risk (Buxton et al., 1999; Kuck et al., 2000; Connolly et al., 2000a,b; Moss et al., 2002; Bardy et al., 2005). As ICDs can avoid SCD but cannot affect the underlying cardiac substrate, the prolonged lifespan enjoyed by patients with significant heart disease is thus shifting the clinical burden to the resulting increase in heart failure events (Sears et al., 2006; Mishkin et al., 2009) and to the possibility of repeated shocks (Barnay et al., 2007). Reported rates of appropriate ICD shocks range from 60% in the 3-year secondary prevention AVID study (Anderson et al., 1999) to 20% in the 2-year primary prevention MADIT II trial (Moss et al., 2002).

As many patients who receive a shock develop some form of psychological distress in the aftermath (Sears and Kirian, 2010), the possible relation between ICD shocks and psychological distress/disorders or reduced QoL was assessed with particular attention. Investigations of such relationship were largely confined to descriptive or observational studies because of the clear impossibility to control the shock factor and thus to use randomized designs. Despite these limitations, an amount of studies attempted to test the hypothesis that ICD shocks are responsible for the occurrence of psychological distress/disorders and the reduction of QoL in ICD patients. However, findings were promptly discordant (Sears et al., 1999; Burke et al., 2003) and the supposed negative effect of ICD shock on patients' QoL and psychological health is still an object of debate (Pedersen and Van Den Broek, 2008; Pedersen et al., 2010b).

In order to examine whether such mixed findings might depend on design and methodological differences, we reviewed and critically appraised all quantitative studies that statistically assessed the effect of ICD shocks on psychological variables such as QoL, anxiety, depression, psychological stress or well-being and post-traumatic stress disorder (PTSD) in patients implanted with an ICD for primary and secondary prevention.

## Methods

### Study eligibility criteria

#### Types of studies

Quantitative studies that statistically assessed the association between ICD shock and psychological outcomes were included. Qualitative and single-case or case-series reports were not considered. Studies were selected irrespective of designs, aims, hypotheses, time from ICD implantation and length of follow-up. Studies that explicitly assessed the causal effect of psychosocial factors on ICD shock occurrence were not considered.

#### Types of participants

Patients of age ≥18 implanted with an ICD for primary or secondary prevention of SCD were considered. No restriction was made on patients' clinical and demographic characteristics with the exception of age. Young patients of age <18 were not considered.

#### Types of intervention

Only automatic ICD shock therapy was considered. No restriction was made on appropriateness (both appropriate and misappropriate shocks were considered), duration, electric power and the absolute or relative number of delivered shocks (isolated shocks, electric storms and shock clusters were considered).

#### Types of outcome measures

Only valid and reliable standardized measures of psychological and quality of life outcomes were considered. Anxiety, depression, PTSD and health-related quality of life measures were specified in advance and documented in the review protocol. However, no restriction was made on any other psychological outcomes that were measured in a valid and reliable manner.

### Report eligibility criteria

Only English articles that were published in indexed journals were considered. Abstracts, letters, unpublished data and gray literature in general were not searched nor considered.

### Data sources

A comprehensive search of English articles that were published between 1980 and 30 June 2013 was applied to the following electronic databases: PubMed, EMBASE, NHS HTA database, PsycINFO, Sciencedirect and CINAHL. Since the first ICD implantation took place in 1980, it was unnecessary to search records predating that year. Bibliographies of included studies were screened for further references.

### Literature search

A two-step search strategy was used. We arbitrarily defined four time intervals (1980–1996, 1997–2003, 2004–2007, 2008–2011) and randomly assigned each of them to one of four independent reviewers who had been previously instructed about the search protocol. We first searched online databases for the following terms in article titles or abstracts: ICD, implant^*^, defib^*^, cardiover^*^, (internal near defib^*^), (internal near cardiover^*^), (implant^*^ near cardiover^*^), (implant^*^ or internal), (cardiac near defib^*^), (implant^*^ and defib^*^), (internal and defib^*^), (cardiac and defib^*^). We recorded results in a reference database (EndNote X2, the Thomson Corporation). The second step refined the first search by filtering for the following terms: mood, quality of life, QOL, health, health-related quality of life, adjust^*^, psych^*^, anx^*^, depress^*^, stress, well-being.

### Study selection

Studies identified by the whole search strategy were assessed for inclusion through three stages. First, three independent reviewers (GMM, RP, and GC) screened titles and abstracts of papers to exclude irrelevant records. Full-texts of remaining papers were obtained and assessed against eligibility criteria by the same independent reviewers at the second stage. Any differences in opinion were resolved through discussion with a forth independent reviewer at the third stage.

### Data collection

All included studies were randomly and equally assigned to three review authors (GMM, RP, SC) who independently extracted and coded data in accordance to a refined sheet. Information was extracted on: (1) design and aim of study, (2) ICD indication (primary, secondary or both) and programming (only shock or shock and pacing); (3) demographic characteristics of participants implanted with an ICD (age and sex); (4) inclusion and exclusion criteria; (5) number of participants included in the analysis and lost to follow-up; (6) shock therapy; (7) timing of psychological assessments and length of follow-up; (8) outcome measures; (9) statistical analysis; (10) results.

### Risk of bias assessment

The Cochrane data collection form for non-randomized studies and the Newcastle-Ottawa scale for assessing quality of cohort studies were used to assess risk of bias and to ascertain the validity of studies. Both templates were retrieved from the Cochrane Handbook for Systematic Reviews of Interventions, Chapter 13, Supplementary Material (retrieved at www.cochrane.org/training/cochrane-handbook). The same three review authors (GMM, RP, and GC) who extracted data determined also the adequacy of: (1) ascertainment of shock exposure; (2) demonstration that outcomes of interest was not present before ICD implantation; (3) control for confounding; (4) assessment of outcomes (self-report or interview); (5) validity and reliability of outcome measures; (6) completeness of data set. Furthermore, the review authors appraised the method used for identifying relevant confounders and the method used for controlling relevant confounders.

### Data analysis

Wide methodological differences were promptly observed across studies during the review process. The feasibility of standardizing individual study results and combining them in a meta-analysis was thus discussed several times during the first intermediate meetings and no consensus was established before the middle of the review process when we decided not to perform any meta-analysis. As reported extensively in the results section, included studies differ in many characteristics, in particular analytical and operationalizing methods, and statistical combination of data from two or more similar studies in a meta-analysis may be neither necessary nor desirable (Liberati et al., 2009). Hence, the extracted data were only qualitatively analyzed and tabulated. Despite the danger of poor validity associated to quasi-quantitative methods such as vote counting, we decided also to perform subgroup analyses in order to explore if significant results depend on the methodological factors we specified in advance. Publication bias or selective reporting bias were not systematically assessed because we did not specify this possibility before starting the review and, even if we observed some clues of selective reporting in a number of articles, we decided not to investigate further.

## Results

### Study selection

A total of 60 studies were identified for inclusion in the review (Table 1). The first-step search of electronic databases provided a huge amount of records that were then drastically reduced with the filtering for the second-step search terms. Of these records, a large part was discarded in accordance to the report eligibility criteria and because, after screening the abstracts, it appeared that these studies clearly did not assess a statistical association between ICD shock and the outcomes of interest. No further article was discarded after examining the full text of the remaining 60 records. No additional study was identified by checking the references of relevant papers, by searching for studies that have cited these papers or by contacting the principal authors of the field.

**Table 1:** **Characteristics of studies**.

**Source**	**Indication**	**Sample^1^**	**Age (mean ± SD)**	**Age (range)**	**Gender (% females)**	**Inclusion criteria**	**Exclusion criteria**
Keren et al., 1991	Secondary	18	62	Not reported	0%	Not reported	History of organic brain syndrome
							Psychiatric hospitalizations
							Anxiolytic, antidepressant or neuroleptic medications at the time of the initial clinical evaluation
Morris et al., 1991	Secondary	20	60.9	Not reported	35%	Not reported	Not reported
Luderitz et al., 1993	Unclear	57	59 ± 13	Not reported	12%	Third-generation ICD ATP device	Not reported
Dougherty, 1995	Secondary	15	57	Not reported	13.4%	Cardiac arrest from primary VF	Physiologically or neurologically compromised
							AMI in conjunction with SCA or a cardiac arrest for reasons not related to cardiovascular causes
Chevalier et al., 1996	Primary	32	54.5	22–73	12.5%	Not reported	Not reported
Burgess et al., 1997	Not declared	25	65	29–80	0%	Not reported	Not reported
Herrmann et al., 1997	Both	63	61 ± 13	Not reported	21%	Not reported	Not reported
Heller et al., 1998	Primary	58	64 ± 11	37–84	28%	Not reported	Not reported
Dunbar et al., 1999	Both	163	59.6 ± 13	24–85	17%	Intact cognitive status	History of psychiatric disorder
Dunbar et al., 1999	Both	176	59.8 ± 13	25–85	18%	Intact cognitive status	History of psychiatric illness requiring medication, psychotherapy or hospitalization
Goodman and Hess, 1999	Not declared	25	65	29–80	0%	Not reported	Not reported
Herbst et al., 1999	Not declared	49	65 ± 11.7	Not reported	12%	Not reported	Major cardiac surgery (i.e., heart transplantation or CABG) or pacemaker implantation
							LVEF < 35%
							NYHA Class < III
Namerow et al., 1999	Primary	262	63.6 ± 9.2	Not reported	15%	Scheduled CABG	Participation in the enrolling center's ICD support group meetings
						LVEF <0.36	
						Abnormal signal-averaged ECG	
Pauli et al., 1999	Both	61	55.7 ± 9	25–65	20%	Not reported	Not reported
Duru et al., 2001	Not declared	76	57 ± 13	Not reported	15.8%	Not reported	Not reported
Godemann et al., 2001	Secondary	72	69.1 ± 10.4	Not reported	13.9%	Intellectual and physical fit for participation	Not reported
Irvine et al., 2002	Secondary	86	64.4 ± 8.6	Not reported	12.1%	CISD	Not reported
Schron et al., 2002	Secondary	373	64.3 ± 10.5	Not reported	18.7%	AVID	Not reported
Wallace et al., 2002	Secondary	58	67	Not reported	24%	Not reported	Not reported
Kamphuis et al., 2002	Secondary	133	55.24 ± 13.7	Not reported	26.3%	Out-of-hospital cardiac arrest	Not reported
Kamphuis et al., 2003	Secondary	132	55.24 ± 13.7	Not reported	26.5%	Not reported	Not reported
Newman et al., 2003	Primary	150	62 ± 12	Not reported	27%	2 symptomatic AF or atrial flutter episodes in the 3 months before implantation and to have failed at least 1 antiarrhythmic drug because of inefficacy or toxicity	History of sustained ventricular tachyarrhythmias or class IV heart failure
Strickberger et al., 2003	Primary	51	58 ± 11	Not reported	33%	NIDCM	Syncope, pregnancy, a contraindication to Amiodarone or defibrillator therapy or concomitant therapy with a Class I antiarrhythmic drug
						LVEF = 0.35 asymptomatic NSVT	
						NYHA functional class I to III	
Godemann et al., 2004a	Secondary	90	59.5 ± 11.1	Not reported	13.3%	Intact cognitive status	ICD implantation <1 year previously
Godemann et al., 2004b	Secondary	93	59.7 ± 11.2	29–81	14%	Intact cognitive status	ICD implantation <1 year previously
Pedersen et al., 2004	Secondary	182	62 ± 13	Not reported	19%	Not reported	Terminally ill, brain damage, too many missing values on questionnaires
Wathen et al., 2004	Both	55	67 ± 11	Not reported	21%	Not reported	Hypertrophic cardiomyopathy, long-QT syndrome or Brugada syndrome
Carroll and Hamilton, 2005	Secondary	59	60	21–84	28.8%	Intact cognitive status	Not reported
Pedersen et al., 2005	Secondary	182	62 ± 13	Not reported	19%	Not reported	Terminally ill, brain damage, too many missing values on questionnaires
Sears et al., 2005	Secondary	60	65.3	22–89		Not reported	Cognitive impairment
Bilge et al., 2006	Both	91	53 ± 14	18–86	13.2%	Not reported	Significant psychiatric illness and recent ICD implantation (<3 months)
Cuculi et al., 2006	Both	55	61.6	30–81	23.3%	Recalled and potentially flawed ICD	Not reported
Leosdottir et al., 2006	Not reported	41	61.8 ± 14.2	25–85	31.7%	Not reported	Major mental or physical disabilities
Luyster et al., 2006	Both	100	67.9 ± 11.7	35–85	19%	Not reported	Not reported
Crossmann et al., 2007	Both	35	57 ± 6.3	35–65	14%	LVEF <0.36	Participation in the enrolling center's ICD support group meetings
						Abnormal signal-averaged electrocardiogram	
Passman et al., 2007	Primary	227	59 ± 14	Not reported	27%	LVEF ≤ 35% not caused by CAD History of symptomatic heart failure Either non-sustained ventricular tachycardia or 10 or more premature ventricular depolarizations per hour	Not reported
Pedersen et al., 2007	Secondary	154	58.5 ± 12.5	Not reported	18.8%	Not reported	Life expectancy <1 year, history of psychiatric illness other than affective/anxiety disorders, on the waiting list for heart transplantation
Piotrowicz et al., 2007	Primary	390	Unclear	Not reported	Unclear	MADIT-II	Not reported
Sossong, 2007	Both	90	65.4 ± 10.6	36–88	22.2%	ICD for at least 2 months since implantation	Not reported
						Intact cognitive status	
Johansen et al., 2008	Secondary	610	62.4	18–85	18%	Not reported	First ICD implant within the last 3 months, HTX, death or ICD removed, cognitive impairment, overall insufficient data quality, procedural error
Ladwig et al., 2008	Both	147	59.9 ± 13	Not reported	15%	Time since implantation longer than 3 months	Not reported
						Rapid onset of the CHD condition	
Mark et al., 2008	Primary	816	59.9 ± 11.9	Not reported	22.9%	NYHA chronic and stable class II or III congestive heart failure	Not reported
						LVEF ≤ 35%	
Pedersen et al., 2008a	Secondary	566	61.9 ± 14.3	18–85	18%	Not reported	First ICD implant within the last 3 months, HTX, death or ICD removed, cognitive impairment, overall insufficient data quality, procedural error
Pedersen et al., 2008b	Both	176	59	Not reported	80.7%	LVEF <0.36	Life expectancy <1 year, history of psychiatric illness other than affective/anxiety disorders, on the waiting list for heart transplantation
						Abnormal signal-averaged electrocardiogram	
Van Den Broek et al., 2008	Both	308	62.6 ± 10.1	Not reported	18%	Not reported	Not reported
Jacq et al., 2009	Both	65	59.8 ± 14.8	Not reported	13.8%	Not reported	Previous medical or surgical problem at the time of interview
Noyes et al., 2009	Primary	601	64.6	Not reported	17%	Prior AMI and a LVEF ≤ 0.30	Experience of an ICD shock before baseline HRQOL data collection
Pedersen et al., 2009	Secondary	557	61.9 ± 14.3	Not reported	18.1%	Not reported	First ICD implant within the last 3 months, HTX, death or ICD removed, cognitive impairment, overall insufficient data quality, procedural error
Spindler et al., 2009	Secondary	535	61.5 ± 14.4	Not reported	18.1%	Not reported	First ICD implant within the last 3 months, HTX, death or ICD removed, cognitive impairment, overall insufficient data quality, procedural error
Thomas et al., 2009	Primary	57	59.8 ± 11.8	Not reported	18%	NYHA class II or III	History of ventricular arrhythmias or cardiac arrest.
						LVEF ≤ 35%	
Van Den Broek et al., 2009	Both	165	62.1 ± 10.6	Not reported	12.7%	Not reported	Cognitive impairment (e.g., dementia), severe comorbidities (e.g., cancer)
Kapa et al., 2010	Both	223	66 ± 12	Not reported	17.9%	Not reported	Not reported
Pedersen et al., 2010a	Both	348	57.7 ± 12.1	Not reported	21%	Not reported	Life expectancy <1 year, history of psychiatric illness other than affective/anxiety disorders, on the waiting list for heart transplantation
Redhead et al., 2010	Secondary	100	69	41–88	17%	ICD already implanted over a 3-year period	Not reported
Suzuki et al., 2010	Both	90	57 ± 16	Not reported	28%	new implantation of ICD or CRT-D devices, an existing ICD/CRT-D, upgrade from ICD to CRT-D, generator replacement, electrical storm or acute decompensated heart failure	Not reported
Versteeg et al., 2010	Secondary	300	57.9 ± 12	Not reported	19.7%	MIDAS	Not reported
Dickerson et al., 2010	Both	76	62.4 ± 11.5	32–84	23.8%	Not reported	History of acute psychiatric disorders
Habibovic et al., 2012	Both	395	62.8 ± 10.3	Not reported	19%	Not reported	Cognitive impairments (e.g., dementia), psychiatric history (other than affective disorders), life-threatening comorbidities (e.g., cancer), life expectancy <1year
Pedersen et al., 2011	Both	284	61.2 ± 10.2	Not reported	21.1%	Not reported	Significant cognitive impairments (e.g., dementia), life-threatening comorbidities (e.g., cancer), history of psychiatric illness other than affective/anxiety disorders
Von Känel et al., 2011	Both	107	57.2 ± 14.2	Not reported	38.3%	Time since implantation longer than 3 months	Not reported

1 *Number of ICD patients whose data were analyzed*.

### Characteristics of included studies

#### Designs

Study designs were coded considering only the part of study in which an association between ICD shock and outcomes of interest was assessed. According to criterion, 32 studies out of 60 were classified as cross-sectional, 27 as prospective and 1 as randomized controlled trial (RCT). Hence, prospective cohort studies that evaluated the effect of shock cross-sectionally (e.g., Mark et al., 2008) were coded as cross-sectional. Only cohort studies that assessed the effect of ICD shock on change in psychological variables and quality of life along time were considered prospective. Six of the included studies are randomized controlled trials (Namerow et al., 1999; Irvine et al., 2002; Schron et al., 2002; Strickberger et al., 2003; Wathen et al., 2004; Mark et al., 2008) but three out of them were classified as cross-sectional (Namerow et al., 1999; Strickberger et al., 2003; Mark et al., 2008) and two as prospective (Irvine et al., 2002; Schron et al., 2002) because assessment of the shock effect was a sub-analysis performed only on patients randomized to the ICD condition. Only the PainFREE Rx II trial (Wathen et al., 2004) was coded as RCT because the ICD shock was partially manipulated. In fact, patients with ICDs were randomized into two treatment conditions that differed only for the delivering of shock therapy or anti-tachycardia pacing.

#### Participants with ICDs

The included studies vary a lot with respect to sample sizes. The study with the smaller sample involved 15 ICD patients and has a prospective design (Dougherty, 1995), while the study with the larger sample included 816 ICD patients and was coded as cross-sectional although it is an RCT comparing amiodarone vs. ICD in heart failure patients (Mark et al., 2008). Considering only patients with an ICD whose data were included in statistical analyses and contributed to results, the whole number of participants considered in this review is 10558. The average of the mean ages of patients across the included studies is 61.2 with a standard deviation (SD) of 3.6 (range: 53–69.1), while the average of the relative SDs is 12.1 (range: 6.3–16). Patients included in the studies were mainly males. Percentages of females varied from 0% (Keren et al., 1991; Burgess et al., 1997; Goodman and Hess, 1999) to 81% (Pedersen et al., 2008b) with a mean of only 20% (SD 10.9%).

#### ICD indication

As expected, studies that involved only patients with a secondary ICD indication are more frequent than studies that recruited only patients with a primary ICD indication (22 vs. 10). Samples were heterogeneous (both patients with a primary indication and patients with a secondary prevention were recruited) in 22 studies, while in 6 papers no information about ICD indication was reported and relative studies were thus not classified (see Table 1 for details).

#### ICD shock therapy

Twenty-seven studies operationalized number of ICD shocks in a dichotomized variable with patients who received 1 or more shocks classified in one category and patients who did not receive any shock assigned to the other one. Across 22 out of 27 studies that operationalized ICD shocks in this manner (no shock vs. ≥1 shocks), 38.5% of patients received at least 1 shock on average. The smallest percentage of patients who received 1 or more shocks (4.2%) was found in the study of Van Den Broek et al. (2009), while the higher (64%) was found in the study of Crossmann et al. (2007), followed by Jacq et al. (2009) and Bilge et al. (2006) with 61.5% shocked patients. In five of the articles describing the studies that we classified in this category (no shock vs. ≥1 shocks), data about percentage of patients who received 1 or more shock from their ICD were lacking (Keren et al., 1991; Kamphuis et al., 2002; Wathen et al., 2004; Cuculi et al., 2006; Piotrowicz et al., 2007). Indeed, some articles reported only the number, the mean or the median of ICD shocks delivered during the study period. Furthermore, we found that two articles classified in this category (no shock vs. ≥1 shocks) described two studies whose aims and hypotheses were different but shared the same sample (Pedersen et al., 2004, 2005). Two studies operationalized number of ICD shocks in a dichotomized variable with patients who received 5 or more shocks classified in one category and patients who received between 0 and 4 shocks assigned to the other one. In Luderitz et al.'s study (1993), 57.9% of ICD patients received 5 or more shocks during a 12-month follow-up, while in the Von Känel et al.'s study (2011), 8.4% received 5 or more shocks before baseline assessment (24.4 ± 20.7 months post ICD-implantation) and 19.3 % received 5 or more shocks between the baseline and the end of follow-up (65.5 ± 27.4 months post ICD-implantation). Nine studies categorized ICD shocks in multiple groups and eight different categorizations were used. Three studies out of them created an extreme group of patients who had received ten or more shocks (Herrmann et al., 1997; Ladwig et al., 2008; Suzuki et al., 2010), while three studies grouped also patients who had received electrical storms (Kapa et al., 2010; Redhead et al., 2010; Suzuki et al., 2010). Three studies operationalized ICD shocks in units of time. Morris et al. (1991) divided the number of delayed ICD shocks by length of follow-up (in months) to generate a frequency rate per unit of time; Jacq et al. (2009) divided the number of shocks received since implantation by the time elapsed since implantation (ratio shock) in order to take into account the significant difference in time elapsed since implantation between participants who did or did not experience ICD shock; Pauli et al. (1999) calculated the relative number of ICD shocks per year. Finally, six studies calculated the number of ICD shocks that were delivered within a fixed length of time or since last assessment (Kamphuis et al., 2003; Bilge et al., 2006; Mark et al., 2008; Noyes et al., 2009; Dickerson et al., 2010; Suzuki et al., 2010). In all the other studies, the absolute number of ICD shocks that each patient received was considered for the analysis.

#### Outcomes

The most prevalent outcomes are measures of anxiety, depression and health-related quality of life. In particular, anxiety was measured in 36 studies, depression in 30 studies and health-related quality of life (both mental and physical) in 29 studies. Anxiety and depression were mostly measured with self-report questionnaires. In only three studies (two of them used also a self-report questionnaire) anxiety was assessed with a clinical interview (Van Den Broek et al., 2008, 2009; Jacq et al., 2009), while depression was evaluated with a diagnostic interview in only one study (Jacq et al., 2009). With respect to the self-report measure of anxiety as an outcome of ICD shocks, the Hospital Anxiety and Depression Scale (HADS) was the most used psychometric questionnaire (13 studies out of 35, i.e., the total number of studies that used a self-report measure of anxiety, used the HADS). The second most used measure is the Spielberger State-Trait Anxiety Inventory (STAI), which was used in ten studies. The remaining self-report questionnaires that were used to measure anxiety are the Hamilton Anxiety Scale (1 study), the Beck Anxiety Inventory (1 study) and the anxiety index of the Symptom Checklist 90 (1 study). The Hamilton Rating Scale for Anxiety was used in two of the three studies that assessed anxiety with a clinical interview. Differently, Jacq et al. (2009) used the Mini International Neuropsychiatric Interview. With respect to the self-report measure of depression as an outcome of ICD shocks, the Hospital Anxiety and Depression Scale (HADS) was again the most used psychometric questionnaire (13 studies out of 30, i.e., the total number of studies that used a self-report measure of depression, used the HADS). The second most used measure is the Beck Depression Inventory (version 1 or 2) which was used in 6 studies. The remaining self-report questionnaires that were used to measure depression are the Zung Self-Rating Depression Scale (1 study), the Centre for Epidemiologic Studies Depression Scale (1 study) and the depression index of the Symptom Checklist 90 (1 study). The only study that assessed depression symptoms with a clinical interview used the Mini International Neuropsychiatric Interview (Jacq et al., 2009). General mental disorders were assessed in four studies (Morris et al., 1991; Chevalier et al., 1996; Godemann et al., 2001, 2004a). All of them used a semi-structured psychiatric interview according to the DSM-III-R criteria. Health-related quality of life was mostly measured with the SF-36 (15 studies) and the SF-12 (4 studies). Few other studies used the Health Utility Index 3 (Noyes et al., 2009), the Health Complaints Scale (Van Den Broek et al., 2009), the RAND-36 Health Survey (Kamphuis et al., 2002, 2003), the Ferrans and Powers Quality of Life Index (Carroll and Hamilton, 2005; Sossong, 2007; Dickerson et al., 2010), the General Health Questionnaire and the Icelandic Quality of Life Questionnaire (Leosdottir et al., 2006), the RAND-38 Mental Health Inventory and the Nottingham Health Profile (Irvine et al., 2002), and the Quality of Well Being Schedule (Strickberger et al., 2003). Further psychological outcomes are Post-Traumatic Stress Disorder (PTSD) or PTSD symptomatology, ICD acceptance and ICD concerns. PTSD was evaluated in five studies. The Impact of Event Scale-R was used in three studies (Ladwig et al., 2008; Kapa et al., 2010; Von Känel et al., 2011), while the Posttraumatic Stress Diagnostic Scale was administered in the other ones (Versteeg et al., 2010; Habibovic et al., 2012). ICD acceptance was analyzed as an outcome of ICD shocks in three studies (Pedersen et al., 2008a; Spindler et al., 2009; Keren et al., 2011). The Florida Patient Acceptance Survey was used in all of them. Finally, ICD concerns were assessed as an outcome of ICD shocks in two studies (Spindler et al., 2009; Van Den Broek et al., 2009). The ICD Concerns questionnaire was used in both ones.

#### Timing of outcome assessment and follow-up

Included studies vary a lot with respect to the timing of outcome assessment and follow-up. The first sharp distinction concerns study design. However, even considering cross-sectional and prospective studies separately, a large amount of variability remains in each category. In real cross-sectional studies, in which patients were assessed only once, a great heterogeneity in time from ICD implantation was observed both within and between studies. For example, the average of the mean times from ICD implantation across the 19 cross-sectional studies that reported time data on a continuous scale is 32 months with a SD of 18.2 (range: −60). The briefer mean time from ICD implantation was found in Namerow et al.' study (1999), while the longer one was found in Pedersen, Spindler, Johansen and Mortensen study (2009). In Jacq et al.'s study (2009), mean time from ICD implantation was divided between patients who received 1 or more shocks (37.4 months ±31.9) and patients who did not receive any shock (17.9 months ±16), while in 2 studies (Bilge et al., 2006; Redhead et al., 2010) patients were divided into multiple sub-groups according to fixed time intervals. In prospective studies, in which patients were assessed at least twice along the follow-up (repeated measures), differences and heterogeneities were observed in four factors: (1) baseline assessment (before ICD implantation or after ICD implantation); (2) time before ICD implantation; (3) timing of repeated measurements from ICD implantation; (4) length of follow-up. Baseline was clearly assessed before ICD implantation in 14 studies, but in only 3 out of them the baseline time-point was explicitly reported, i.e., 1 day before ICD implantation (Pedersen et al., 2007, 2008a, 2010a). However, these 3 studies are not independent because patients who comprised the three samples participated in the same study (MIDAS—Mood and personality as precipitants of arrhythmia in patients with an ICD: A prospective Study). Baseline was assessed before ICD implantation also in other 3 studies but not for all participants, some of whom were evaluated just after the implantation before hospital discharge (Dunbar et al., 1999; Irvine et al., 2002; Suzuki et al., 2010). Baseline was clearly assessed after ICD implantation in 8 studies but the timing of first assessment was highly heterogeneous both between and within them. For example, in some studies patients were evaluated few days after ICD implantation or at hospital discharge, while in other studies patients were firstly assessed after months from surgery. Finally, if baseline assessment was performed before or after the ICD implantation was impossible to establish in three studies because the respective articles do not report sufficient information. Prospective studies are quite heterogeneous also with respect to the number and timing of repeated measurements from ICD implantation and length of follow-up. For example, in only 19 out of 28 studies patients were followed for at least 12 months (see Table 2 for details).

**Table 2:** **Study methods**.

**Source**	**Study name**	**Design**	**Timing of outcome assessment**	**Self-report measures**	**Outcomes**	**Shock operationalization**	**Shock analysis**
Keren et al., 1991		Cross-sectional	18 months post-ICD implantation (range 4–34)	Self-report: - STAI-Y^10^ - BDI^11^ - *Ad-hoc* questionnaire	State-anxiety	Dicothomized (yes/no)	Shock as grouping variable
					Trait-anxiety		
					Depression		
					ICD Experiences		
Morris et al., 1991		Cross-sectional	7.5 months post-ICD implantation (range 3–21)	Semi-structured psychiatric interview (DSM-III-TR)	Mental disorders	Shock ratio (Shock frequency devided by lenght of follow-up)	Shock ratio as test variable Mental disorder as grouping variable (3 groups)
Luderitz et al., 1993		Prospective	Before and 1, 3, 6, 12 months post-ICD implantation	Self-report: - STAI-Y^10^ - *Ad-hoc* questionnaire	State-anxiety ICD appraisal	Dicothomized (0–4/≥5)	Shock as grouping variable
Dougherty, 1995		Prospective	At hospital discharge and 6, 12 months after	Self-report: - POMS^12^ - STAI-Y^10^ - the Distancing Subscale of the Ways of Coping Checklist-Revised - The Dyadic Adjustment Scale - The F-COPES	State-anxiety-tension	Dicothomized (yes/no)	Shock as grouping variable
					Depression		
					Anger		
					Stress		
					Denial		
Chevalier et al., 1996		Cross-sectional	25 ± 1.6 months post-ICD implantation (range 1–54)	Interviewer: - Diagnostic interview (DSM-III-TR)	Depression symptoms	Dicothomized (yes/no)	Shock as grouping variable
				Self-report: - Hamilton Anxiety scale - BDI^11^ - MMPI^13^ - *Ad-hoc* ICD-QoL	Anxiety symptoms		
					ICD-related QoL		
					Mental disorders		
Burgess et al., 1997		Cross-sectional	Unclear	Self-report: - SCL-90-R - demographics questionnaire (premorbid medical and psychiatric self-report health histories. Life-style changes)	Psychological distress	Shock frequency	Shock as predictor variable
Herrmann et al., 1997		Cross-sectional	510 ± 408 days since implantation	Self-report: - HADS^14^ - Quality-of-Life Profile for the Chronically ill (PLC) - Unstandardized items dealing with patients' attitudes toward the ICD	Anxiety symptoms	Categorized (0–4/5–9/≥10)	Shock as grouping variable
					Depression symptoms		
					QoL		
Heller et al., 1998		Cross-sectional	20 ± 14 months post-ICD implantation	Self-report: - BDI^11^ - STAI-Y^10^ - Cook-Medley subscale of the MMPI- questions examining attitudes toward the ICD experience and cardiac illness	Emotional states	Dichotomized (0–4/5–9)	Shock as predictor variable
Dunbar et al., 1999		Prospective	Before (for 7% soon after implantation) and 1, 3 months post-ICD implantation	Self-report: - Life Orientation Test (LOT) - Threat and Challenge subscales from the Meaning in Illness Questionnaire (MIQ) - The symptom and fear components from the ICD Concerns Questionnaire - The Jalowiec Coping Scale (JCS) - POMS^12^ - Heart Failure Functional Status Inventory (HFFSI)	Total mood disturbance	Shock frequency	Shock as predictor variable
Dunbar et al., 1999		Prospective	Before and 1, 3, 6, 9 months post-ICD implantation	Self-report: - POMS^12^ - STAI-Y^10^ - the Distancing Subscale of the Ways of Coping Checklist-Revised - The Dyadic Adjustment Scale - The F-COPES	Emotional states	Shock occurrence	Shock as within-subject factor
Goodman and Hess, 1999		Cross-sectional	Unclear	Self-report: - SCL-90-R	Anxiety symptoms	Shock frequency	Shock as predictor variable
				- demographics questionnaire (premorbid medical and psychiatric self-report health histories)	Depression symptoms		
Herbst et al., 1999		Cross-sectional	31.2 ± 16.8 months post-ICD implantation	Self-report: - SF-36 - Brief Symptom Inventory - demographics questionnaire (premorbid medical and psychiatric self-report health histories)	QoL	Dicothomized (yes/no)	Shock as grouping variable
					Psychological distress		
Namerow et al., 1999	CABG Patch Trial	RCT	6 months after CABG surgery	Self-report: - SF-36 - demographics questionnaire (premorbid medical and psychiatric self-report health)	QoL	Dicothomized (yes/no)	Shock as grouping variable
		Cross-sectional					
Pauli et al., 1999		Cross-sectional	22.8 ± 19.2 months post-ICD implantation (range 2–89)	Self-report: - AICD-questionnaire (anxiety related to future shocks) - The ACQ (catastrophizing cognitions) - The BSQ (anxiety of bodily symptoms) - STAI-Y^10^ - BAI^15^ - BDI^11^	Shock-related anxiety	Shocks frequency	Shock as predictor variable
						Shock ratio (Shock frequency devided by lenght of follow-up) Dichotomized (yes/no)	Shock as fixed factor
Duru et al., 2001		Cross-sectional	≥6 months post-ICD implantation (2.3 years on average)	Self-report: - SF-36 - HADS^14^ - *Ad-hoc* questionnaire (perceptions of ICD)	Anxiety symptoms	Dicothomized (yes/no)	Shock as grouping variable
					Depression symptoms		
					ICD appraisal		
Godemann et al., 2001		Cross-sectional	3.4 ± 1.8 years post-ICD implantation	Interviewer: - Semi-structured interview (DIPS)	Mental disorders	Shock frequency	Shock as predictor variable
				Self-report: - SCL-90-R			
Irvine et al., 2002	CISD^9^	RCT	Before or soon after randomization and 2, 6, 12 months post-ICD implantation	Self-report: - Rand Corporation's 38-item Mental Health Inventory - Nottingham Health Profile	QoL	Categorized (0/1–4/≥5)	Shock as fixed factor
		Prospective					
Schron et al., 2002	AVID^8^	RCT	Before randomization and 3, 6, 12 months after randomization	Self-report: - SF-36 - Patient concerns checklist - The cardiac version of the QoL index	QoL	Dichotomized (yes/no and <3/≥3)	Shock as fixed factor
		Prospective					
Wallace et al., 2002		Cross-sectional	12–24 (70.6%), 25–36 (27.4%) and 37–48 (2%) months post-ICD implantation	Self-report: -State Trait Personality Inventory - Interpersonal Support Evaluation List - Disease-specific QoL AVID checklist - SF-12	QoL	Shocks frequency	Shock as predictor variable
Kamphuis et al., 2002		Prospective	Few days before and 1, 6, 12 months post-discharge	Self-report: - Rand 36-item Health Survey - The Heart Patients Psychological Questionnaire (HPPQ) - CES-D^16^ - STAI-Y^10^	QoL	Dicothomized (yes/no)	Shock as fixed factor
					State-Anxiety		
					Depression symptoms		
					Psychological well-being		
Kamphuis et al., 2003		Prospective	after admission (before cardiac evaluation) and 1, 6, 12 months after discharge	Self-report: - RAND-36 - Heart Patient Psychological Questionnaire (HPPQ) - STAI-Y^10^ - CES-D^16^	QoL	Categorized (Shocks in both time intervals/Shocks exclusively during the first 6 months/Shocks exclusively during the last 6 months/No shocks during first year)	Shock as fixed factor
					State-Anxiety		
					Depression symptoms		
					Psychological well-being		
Newman et al., 2003		Prospective	At baseline (?) and 3, 6 months post-baseline	Self-report: - SF-36 - The Symptom Checklist - AF symptoms.	QoL	Categorized (0/1–4/≥5 shocks)	Shock as fixed factor
Strickberger et al., 2003	AMIOVIRT^7^	Cross-sectional	Before and 2 ± 1.3 years post-ICD implantation (range 0.1–4.8 years)	Self-report: - Quality of Well Being Schedule - STAI-Y^10^	State-Anxiety	Dicothomized (yes/no)	Shock as grouping variable
Godemann et al., 2004a		Cross-sectional	3.5 ± 2.0 years post-ICD implantation	Interviewer: - Diagnostic Interview of Psychiatric Disorders -DSM-III-R	Mental disorders	Shock frequency	Shock as predictor variable
				Self-report: - Cognitive coping with shocks			
Godemann et al., 2004b		Cross-sectional	3.4 ± 2.8 years post-ICD implantation (range 1–11)	Self-report: - SF-12 - The Freiburg Questionnaire on Disease Coping (short version) - SCL-90-R	QoL	Shock frequency	Shock as predictor variable
Pedersen et al., 2004	MIDAS^2^	Cross-sectional	55 ± 35 months post-ICD implantation (range 8–132)	Self-report: - HADS^14^ - Type D Personality Scale (DS14) - The Perceived Social Support Scale (PSSS)	Anxiety symptoms	Dichotomized (yes/no)	Shock as predictor variable (direct and interaction effects)
					Depression symptoms		
Wathen et al., 2004	PainFREE Rx II^6^	RCT	Before and 1 year post-ICD implantation	Self-report: - SF–36	QoL	Not applicable	ATP treatment vs. shock treatment
		Prospective					
Carroll and Hamilton, 2005		Prospective	From time of ICD implantation to 1 year after implantation	Self-report: - Ferrans and Powers Quality of Life Index - SF-36 - POMS^12^ - Brodsky ICD Questionnaire	QoLEmotional states	Dicothomized (yes/no)	Shock as grouping variable
					ICD concerns		
Pedersen et al., 2005	MIDAS^2^	Cross-sectional	55 ± 35 months post-ICD implantation (range 8–132)	Self-report: - The ICDC Questionnaire (concerns) - HADS^14^	Anxiety symptoms	Dichotomized (yes/no)	Shock as predictor variable
					Depression symptoms		
Sears et al., 2005		Prospective	During hospitalization, 6–9 and 12–15 months after ICD implantation	Interviewer: - The Schedule for Affective Disorders and Schizophrenia (DSM-IV)	QoL	Dichotomized (yes/no)	Shock as predictor variable
				Self-report: - Interpersonal Support Evaluation List - Short-Form - The Life Orientation Test - STAI-Y^10^ - SF-36 - The Seattle Angina Questionnaire			
Bilge et al., 2006		Cross-sectional	3–6 months (15.4%), 6 months–1 year (2.2%), 1–5 years (68.1%), >5 years (14.3%) post-ICD implantation	Self-report: - HADS^14^	Anxiety symptoms	Dichotomized (Yes/No)	Shock as predictor variable
					Depression symptoms	Shock frequency	
Cuculi et al., 2006		Cross-sectional	Not reported	Self-report: - Brief Symptom Inventory	Psychological distress	Dicothomized (yes/no)	Shock as grouping variable
Leosdottir et al., 2006		Cross-sectional	37.8 ± 28.6 months post-ICD implantation (range 11.6–154.9)	Self-report: - BAI^15^ - BDI^11^ - The General Health Questionnaire - 30-item - The Icelandic Quality of Life Questionnaire (IQL) - ICD Psychosocial Index	Anxiety symptoms	Dicothomized (yes/no)	Shock as grouping variable
					Depression symptoms		
					QoL		
Luyster et al., 2006		Cross-sectional	1.9 ± 1.8 years post-ICD implantation (range 0.07–8.8)	Self-report: - The ENRICHD Social Support Inventory - The Duke Activity Status Index - The Conservation of Resources Evaluation (COR-E) - BDI^11^ - Brief Patient Health Questionnaire - STAI-Y^10^	Depression symptomsTrait-anxiety	Dichotomized (yes/no)	Shock as predictor variable
Crossmann et al., 2007		Prospective	25.5 ± 19.2 months post-ICD implantation (range 1.5–88) and 30.3 months after first assessment (range 29.6–31.2)	Self-report: - The ACQ (catastrophizing cognitions) - The BSQ (anxiety of bodily symptoms) - STAI-Y^10^ - BAI^15^ - The Mobility Inventory (avoidance behavior)	Trait-anxiety	Dicothomized (yes/no)	Shock as grouping variable
					Anxiety symptoms		
					Anxiety related to bodily symptoms		
Passman et al., 2007		Prospective	Baseline (?), 1 and 3 months after randomization and every 3 months thereafter up to 63 months	Self-report: - SF-12 - the Minnesota Living with Heart Failure Questionnaire	QoL	Shock occurrence	Shock as within-subject factor
Pedersen et al., 2007	MIDAS^2^	Prospective	1 day prior to implantation and 3 months post-ICD implantation	Self-report: - Type D Personality Scale (DS14) - SF-36	QoL	Shock frequency	Shock as covariate
Piotrowicz et al., 2007	MADIT-II^3^	Prospective	At baseline (before randomization) and at 12-month follow-up	Self-report: - SF-12	QoL	Dicothomized (yes/no)	Shock as grouping variable
Sossong, 2007		Cross-sectional	15.9 ± 13 months post-ICD implantation (range 2.1–56.1)	Self-report: - The Sossong ICD Knowledge Questionnaire - Mishel Uncertainty in Illness Scale (MUIS-Adult) - Ferrans and Powers Quality of Life Index - Cardiac Version IV	QoL	Shock frequency	Shock as predictor variable
Johansen et al., 2008		Cross-sectional	4.8 years post-ICD implantation (range 0.4–15.9)	Self-report: - HADS^14^ - SF-36 - The Minnesota living with heart failure questionnaire	Anxiety symptoms	Shock frequency	Shock as predictor variable
					Depression symptoms		
					QoL		
Ladwig et al., 2008	LICAD^1^	Cross-sectional	27 ± 21 months post ICD implantation (range 3–142)	Self-report: - Impact of Event Scale-R	PTSD symptoms	Categorized (0/1–4/5–9/≥10)	Shock as crossing variable
Mark et al., 2008	SCD-HeFT^4^	RCT	3, 12, and 30 months post-ICD implantation	Self-report: - SF-36 - Mental Health Inventory 5	QoL	Dicothomized (yes/no) within different time intervals (within 1 month and 2 months before a scheduled QoL assessment and at any time along follow-up)	Shock as grouping variable
		Cross-sectional					
Pedersen et al., 2008a		Cross-sectional	4.7 ± 3.3 years post-ICD implantation (range 0.4–15.9)	Self-report: - the Florida Patient Acceptance Survey	ICD Acceptance	Shock frequency	Shock as predictor variable
Pedersen et al., 2008b	MIDAS^2^	Prospective	1 day prior and 6 months post-ICD implantation	Self-report: - HADS^14^	Anxiety symptoms	Shock frequency	Shock as covariate
					Depression symptoms		
Van Den Broek et al., 2008		Prospective	0 and 3 weeks after ICD implantation and 2 months after	Self-report: - STAI-Y^10^	Anxiety symptoms	Dicothomized (yes/no)	Shock as fixed factorShock as predictor variable
				Interviewer: - Hamilton Rating Scale for Anxiety			
Jacq et al., 2009		Cross-sectional	Shock: 37.4 ± 31.9 months (range 6–44)	Self-report: - HADS^14^ - SF-36	Anxiety symptoms	Dichotomized (Yes/No)	Shock as grouping variable
			No shock: 17.9 ± 16 months (range 6–66)	Interviewer:- Mini International Neuropsychiatric Interview	Depression symptoms	Shock ratio (Shock frequency/time elapsed since implantation)	Shock as crossing variable
					QoL		Shock ratio as correlational variable
Noyes et al., 2009	MADIT-II^3^	Prospective	0, 3, 12, 24, 36 months post-ICD implantation	Self-report: - Health Utility Index 3	QoL	Shock occurrence	Shock as within-subject factor
Pedersen et al., 2009		Cross-sectional	4.9 ± 3.2 years post-ICD implantation	Self-report: - HADS^14^ - The 18-item Florida Patient Acceptance Survey - Type D Personality Scale (DS14)	Anxiety symptoms	Shock frequency	Shock as covariate
					Depression symptoms		
Spindler et al., 2009		Cross-sectional	4.6 ± 3.2 years post-ICD implantation	Self-report: - HADS^14^ - The ICD Concerns questionnaire (8 item) - The 18-item Florida Patient Acceptance Survey - SF-36	Anxiety symptoms	Dicothomized (yes/no)	Shock as covariate
					Depression symptoms		
					ICD concerns		
					Device acceptance		
					QoL		
Thomas et al., 2009	SCD-HeFT/PFOS^5^	Prospective	At entry, 1, 6, 12, 18, and 2 years after ICD implantation	Self-report: - BDI-2 - STAI-Y^10^ - The Social Support Questionnaire-6	State-anxiety	Dicothomized (yes/no)	Shock as predictor variable
					Depression symptoms		
Van Den Broek et al., 2009		Prospective	7.7 ± 6.8 days and 2 months after ICD implantation	Self-report: - STAI-Y^10^ - The 18-item Cardiac Anxiety Questionnaire - The Health Complaints Scale - the ICD-Concerns questionnaire (8 item)	Anxiety symptoms	Dicothomized (yes/no)	Shock as predictor variable
				Interviewer: - The Hamilton Rating Scale for Anxiety	ICD concerns		
					Health complaints		
Kapa et al., 2010		Prospective	Within 2 months after ICD implantation, 6 and 12 months following baseline	Self-report: - HADS^14^ - Impact of Events Scale-Revised - SF-36	PTSD symptoms	Categorized (0/≥1/electrical storm)	Shock as fixed factor
					Anxiety symptoms		
					Depression symptoms		
					QoL		
Pedersen et al., 2010a	MIDAS^2^	Prospective	1 day prior to ICD implantation (baseline) and 10 days, 3 months, 6 months and 1 year post-ICD implantation.	Self-report: - STAI-Y^10^ - the ICD-Concerns questionnaire (8 item) - Type D Personality Scale (DS14) - Multidimensional Scale of Perceived Social Support	State-anxiety	Shock frequency	Shock as predictor variable
					Trait-anxiety		
					ICD concerns		
Redhead et al., 2010		Cross-sectional	6-month “time windows”: 6–12, 12–18, 18–24, 24–30, and 30–36 months post-ICD implantation	Self-report: - HADS^14^ - SF–36	Anxiety symptoms	Categorized (0/≥1/storm)	Shock as crossing variable
					Depression symptoms		
					QoL		
Suzuki et al., 2010		Prospective	Within 7 days before implantation or 8 ± 5 days after admission and 2 years later	Self-report: - Zung Self-Rating Depression Scale	Depression	Dichotomized (yes/no)	Shock as test variable
						Dichotomized (within 6 months /beyond 6 months) Categorized (0, 1–9, ≥10)	Depressed patients vs. non-depressed patients
Versteeg et al., 2010	MIDAS^2^	Prospective	3 and 6 months post-ICD implantation	Self-report: - The Posttraumatic Stress Diagnostic Scale	PTSD symptoms	Shock frequency	Shock as predictor variable
Dickerson et al., 2010		Prospective	Before and 1 and 3 months after ICD implantation	Self-report: - STAI-Y^10^ - the Ferrans and Powers Quality of Life Index, Cardiac Version	State-anxiety	Shock frequency	Shock as test variable
							Comparison of QoL-change patterns
Habibovic et al., 2012		Cross-sectional	0–3 weeks and 18 months post-ICD implantation	Self-report: - STAI-Y^10^ - the Posttraumatic Stress Diagnostic Scale - Type D Personality Scale (DS14)	PTSD symptoms	Shock frequency	Shock as predictor variable
Pedersen et al., 2011	MIDAS^2^	Prospective	0–3 weeks and 12 months post-ICD implantation	Self-report: - STAI-Y^10^ - Type D Personality Scale (DS14)	Chronic anxiety	Shock frequency	Shock as predictor variable
Von Känel et al., 2011	LICAD^1^	Prospective	24.4 ± 20.7 months post ICD-implantation (baseline)	Self-report: - Impact of Events Scale-Revised - Toronto Alexithymia Scale - HADS^14^	PTSD symptoms	Dichotomized (Yes/No and 0–4/≥5)	Shock as predictor variable
			65.5 ± 27.4 months post-implantation (follow-up)	Interviewer: - Peri-traumatic Dissociative Experiences Questionnaire			
			41.1 ± 18.2 months from baseline to follow-up				

1 *Living with an implanted cardioverter defibrillator study*.

2 *Mood and personality as precipitants of arrhythmia in patients with an ICD, A prospective Study*.

3 *Multicenter Automatic Defibrillator Trial-II*.

4 *Sudden Cardiac Death in Heart Failure*.

5 *Sudden Cardiac Death in Heart Failure/Psychosocial Factors Outcome Study in Sudden Cardiac Death*.

6 *Pacing Fast Ventricular Tachycardia Reduces Shock Therapies Trial*.

7 *Amiodarone vs. Implantable Cardioverter-Defibrillator: Randomized Trial in Patients With Non-ischemic Dilated Cardiomyopathy and Asymptomatic Non-sustained Ventricular Tachycardia*.

8 *Antiarrhythmics vs. Implantable Defibrillators*.

9 *Canadian Implantable Defibrillator Study*.

10 *Spielberger State-Trait Anxiety Inventory – Y form*.

11 *Beck Depression Inventory*.

12 *Profile of Mood States Questionnaire*.

13 *Minnesota Multiphasic Personality Inventory*.

14 *Hospital Anxiety and Depression Scale*.

15 *Beck Anxiety Inventory*.

16 *The Center for Epidemiological Studies Depression Scale*.

#### Statistical analysis

Last but not least, studies vary quite a lot with respect to the statistical analyses that were performed to test the effect of ICD shocks on patients' psychological health and quality of life. Clearly, much of this heterogeneity is explained by the ways outcomes and ICD shocks were operationalized and also by study designs. However, two main analytical solutions were identified: (1) classifying patients in two or more shock-groups in accordance with different numerical criteria and testing the simple or adjusted effect of such dichotomized or categorized shock variable by univariate or multivariate analyses and (2) regressing outcome on number of shocks by multivariate regression analyses. Furthermore, in few studies patients were classified in different outcome-groups according to criteria such as psychiatric diagnoses, outcome change patterns or outcome distribution cut-offs and then compared on number of shocks. Finally, in only two studies intra-individual changes from pre-shock to post-shock assessments were analyzed by hierarchical regression models.

The heterogeneity of analytical approaches can be further explained by three factors: (1) the outcome variable scale (dichotomous, dichotomized or continuous); (2) the operationalization of ICD shocks (see previous paragraph) and (3) the number and kind of covariates/predictors that were entered into the statistical models. A fourth factor that pertains only to multivariate regression models concerns the importance of the ICD shock variable within the analysis. In fact, some of the studies that were included in the review did not handle the ICD shock variable as the main explaining factor but treated it as a potential covariate or controlling predictor. In these studies, the leading role was given to other psychological or medical factors (for example, type-D personality, concerns about the ICD, device acceptance and disease severity) and the ICD shock variable was mainly used as a competing predictor in the statistical explanation of patients' psychological distress.

### Risk of bias within studies

All papers that were included in the review were screened in search of some potential biases that could affect the validity of results. In particular, we searched for the systematic biases that can affect the internal validity of cross-sectional and cohort studies. In this kind of non-randomized studies, the major threat to internal validity concerns all the systematic differences that may exist between groups over and beyond the difference determined by the factor of interest and that may confound its effect. One of the methods that can protect against this bias consists in statistically controlling for the effects of all confounding variables that are related to the outcome and/or to the factor. A further method consists in matching subjects between groups according to some variables (for example, age, sex, type of heart disease, LVEF, NYHA Functional Class, etc.) but this procedure was used in only one study (Keren et al., 1991). All the other studies that attempted to reduce the risk of such a bias used the multiple regression method (35 out of 60). However, the number and kind of confounding variables that were selected and controlled for vary significantly across studies. The effect of the ICD shock was indeed adjusted for heterogeneous confounders and this may partially explain why results are discordant. A further major threat to the internal validity of cross-sectional and cohort studies is the presence of the outcome of interest before the occurrence of the event that hypothetically causes it. This bias, when uncontrolled, may affect seriously the causal meaning of an association and, for example, may lead to the wrong conclusion that the ICD shock caused the development of psychological disorders when the reverse was true. The most robust method that may protect against this bias consists in starting the evaluation of patients quite before the ICD implantation and in collecting short-spaced repeated measures along the follow-up. This was fully accomplished in only 18 prospective studies, in which patients were evaluated for the first time few days before surgery. In all the other prospective studies, the baseline was assessed after the ICD implantation. Anyway, for the issue of the review, i.e., the critical appraisal of methods that were adopted in studies on the psychological effect of ICD shock, the most important part of the procedure is clearly the short-spaced timing of repeated measurements that, combined with the hierarchical analysis of intra-individual pre- to post-shock changes, represents for us the best methodology for enhancing the internal validity of cohort studies whose aim is to evaluate the negative effect of ICD shock on patient's health. Another method that was used in few studies consists in evaluating patients retrospectively. However, this approach is prone to biases (e.g., the recall bias and the response shift) that may affect seriously the validity and reliability of patients' responses and that should be avoided. According to the Newcastle-Ottawa checklist, further threats to the internal validity of cross-sectional and cohort studies are the self-reported exposure to the event, the self-reported assessment of outcome, the low validity and reliability of outcome measures and the incompleteness of data set. Except for this last bias, which may seriously affect the validity of results as much as the previous major ones, all the other items were considered minor threats because of their relative low and negligible impact on the validity of results.

### Results of studies and subgroup analysis

Because of the great heterogeneity that was observed in methods across the included studies, a statistical meta-analysis of effects and moderators was deemed unfeasible and was not performed. Further, no attempt was made to describe each study in a narrative manner because of two reasons: (1) the large number of studies that were included and (2) the review aim to focus mainly on methods and to explore cross-sectionally their effects on results. Hence, key methodological features and results of each study were only coded and tabulated (Tables 2, 3). For example, results were coded with 1 when a significant effect of ICD shock was found and with 0 otherwise. We established the statistical significance of effects only on the basis of final results (in studies where both bivariate and multivariate analyses were performed, we considered only the adjusted effects). A series of subgroup analyses according to study design, shock operationalization, shock analysis and multivariate controlling was then performed only on outcomes for which at least 20 studies were available (twenty units were deemed sufficient to test cross-sectional associations between methodological factors and results). Findings are described in the following paragraphs for each outcome of interest.

**Table 3:** **Statistical analyses and results of studies**.

**Source**	**Statistical analysis**	**Anxiety**	**Anxiety interview**	**Depression**	**Depression interview**	**PTSD**	**QoL-mental**	**QoL-physical**	**Psychiatric disorders**	**ICD acceptance**	**ICD concerns**
Keren et al., 1991	*T*-test	0		0							
Morris et al., 1991	ANOVA								1		
Luderitz et al., 1993	*T*-test	1									
Dougherty, 1995	Mann-Whitney U Test	1		0							
Chevalier et al., 1996	*T*-test	0		0					0		
Burgess et al., 1997	Stepwise Multiple Regression	1		1							
Herrmann et al., 1997	ANOVA	0		1			1				
Heller et al., 1998	Multiple logistic regression	1		1							
Dunbar et al., 1999	Hierarchical multiple regression	0		0				0			
Dunbar et al., 1999	Paired *t*-test	0		0							
Goodman and Hess, 1999	Regression	0		0							
Herbst et al., 1999	MANCOVA	1		1			0	0			
Namerow et al., 1999	ANOVA						0	0			
Pauli et al., 1999	Multiple regression MANOVA	0		0							
Duru et al., 2001	ANOVA	0		0			0	0			
Godemann et al., 2001	Logistic regression								1		
Irvine et al., 2002	ANCOVA						1				
Schron et al., 2002	Generalized estimating equations						1	1			
Wallace et al., 2002	Stepwise regression analysis						1				
Kamphuis et al., 2002	MANOVA						0	1			
Kamphuis et al., 2003	MANOVA for repeated measures	1		1			1	1			
Newman et al., 2003	MANOVA for repeated measures						0	0			
Strickberger et al., 2003	*T*-test	0					0				
Godemann et al., 2004a	Multiple regression								1		
Godemann et al., 2004b	Multiple regression						0	0			
Pedersen et al., 2004	Logistic univariate and multivariate regression	0		0							
Wathen et al., 2004	Wilconxon test						1	1			
Carroll and Hamilton, 2005	Mann-Whitney U test	1		0			0	0			
Pedersen et al., 2005	Logistic univariate and multivariate regression	0		0							1
Sears et al., 2005	Hierarchical multiple regression						1	1			
Bilge et al., 2006	Multiple regression	1		0							
Cuculi et al., 2006	Mann-Whitney U Test	0		0							
Leosdottir et al., 2006	*T*-test	0		0			0	0			
Luyster et al., 2006	Hierarchical multiple regression	0		1							
Crossmann et al., 2007	Mann-Whitney U test	0									
Passman et al., 2007	Mixed-effects hierarchical linear regression						1	0			
Pedersen et al., 2007	ANCOVA						0	0			
Piotrowicz et al., 2007	*T*-test						0	1			
Sossong, 2007	Multiple regression						0	0			
Johansen et al., 2008	Multiple logistic regression	1		1				1			
Ladwig et al., 2008	Chi-squared test					0					
Mark et al., 2008	Mann-Whitney U test						1	1			
Pedersen et al., 2008a	Logistic regression									0	
Pedersen et al., 2008b	ANCOVA	1		1							
Van Den Broek et al., 2008	ANCOVA Multiple regression	1	0								
Jacq et al., 2009	Mann-Whitney U test Fisher's exact test Spearman correlation	1	1	1	0	1	1				
Noyes et al., 2009	Logistic and linear regressions (mediation models)						1	1			
Pedersen et al., 2009	ANCOVA	1		0							
Spindler et al., 2009	ANCOVA	1		0			0	0			1
Thomas et al., 2009	Linear mixed models	0		0							
Van Den Broek et al., 2009	Multiple regression		0					1			1
Kapa et al., 2010	MANOVA	1		0		1	0	0			
Pedersen et al., 2010a	Hierarchical, latent class regression models	1									
Redhead et al., 2010	Chi-squared test	1		0			1	0			
Suzuki et al., 2010	Chi-squared test			1							
Versteeg et al., 2010	Logistic regression					1					
Dickerson et al., 2010	ANOVA						0	0			
Habibovic et al., 2012	Multiple linear and logistic regression					0					
Pedersen et al., 2011	Multiple logistic regression	0									
Von Känel et al., 2011	Multiple linear and logistic regression					1					

1 means “significant effect,” while 0 means “non-significant effect.”

#### Anxiety

Patients' anxiety was assessed as an outcome in 35 studies and it was mainly measured by self-report questionnaires. In RCTs that were included in the review, anxiety was never measured. A statistically significant effect of ICD shocks on self-reported anxiety was found in 17 studies, while a significant effect of shocks on interviewer-rated anxiety was found in only 1 study out of 3. Subgroup analyses (Fisher's exact test) did not demonstrate any significant association between the methodological factors and the statistical significance of the shock effect on anxiety.

#### Depression

Patients' depression was assessed as an outcome in 29 studies and, as for anxiety, it was mainly measured by self-report questionnaires. Even depression was never measured in the RCTs included in the review. A statistically significant effect of ICD shocks on self-reported depression was detected in 10 studies, while a non-significant result was found in the only study in which depression was rated by a clinical interview. Subgroup analyses did not demonstrate any significant association between the methodological factors and the statistical significance of the shock effect on depression.

#### Health-related quality of life

Patients' health-related quality of life was assessed as an outcome in 30 studies and was always measured with self-report questionnaires. In most studies, both mental and physical components were evaluated, while in few studies only mental health (4 studies) or physical health (3 studies) were evaluated. With respect to mental health or psychological well-being, a statistically significant effect of ICD shock was found in 12 studies out of 27, while a statistically significant effect of ICD shock on physical health was detected in 11 studies out of 26. Subgroup analyses did not demonstrate any significant association between the methodological factors and the statistical significance of the shock effect on quality of life.

#### Post-traumatic stress disorder (PTSD)

PTSD or PTSD symptoms were assessed as outcomes in five studies and were always measured with self-report questionnaires. A statistically significant effect of ICD shocks was found in 3 studies.

#### Psychiatric disorders

In four studies the effect of ICD shocks was assessed on psychiatric diagnosis of mental disorders and in 3 out of them the effect was statistically significant.

#### ICD acceptance and concerns

ICD acceptance was assessed as an outcome in 1 study but no significant effect of ICD shocks was found, while ICD concerns were measured in 3 studies and the effect of ICD shocks was statistically significant in all of them.

## Discussion

The critical appraisal of the mixed evidence concerning the relationship between ICD shocks and patient-centered outcomes (mainly QoL, anxiety and depression) is the main content of three recently published papers (Pedersen and Van Den Broek, 2008; Pedersen et al., 2010b; Sears and Kirian, 2010). Despite slightly different paradigmatic views on the relative importance of ICD shocks within the group of the numerous factors that may negatively influence the psychological adaptation and well-being of implanted patients, the authors agree that the heterogeneity of designs and methods across studies is most likely to account for the mixed findings.

The quasi-quantitative results of our review do not support this hypothesis. In particular, study design (cross-sectional vs. prospective studies), shock operationalization (the way ICD shocks were operationalized/quantified), shock analysis (the way the effect of ICD shocks was tested) and control for confounding (bivariate vs. multivariate analyses) were examined in vote-counting subgroup analysis, but statistical evidence was null for each of them.

As already noted by Pedersen et al. in a recent viewpoint (2010b), results are mixed even in the subgroup of RCTs. Hence, it seems that the statistical significance of the ICD shock effect on patients' QoL (anxiety and depression were not measured in RCTs) does not depend strictly on sample size. Furthermore, contrary to the hypothesis that a dose-response relationship may exist between the number of shocks and QoL, with only patients experiencing ≥5 shocks being at risk for impaired QoL (Irvine et al., 2002; Pedersen et al., 2010b), studies that categorized the shock variable in classes of increasing shock incidence (e.g., 0–4 vs. 5–9 vs. ≥10 shocks) did not show consistent significant results in any of the outcomes of interest.

However, such null evidence is far from being conclusive. This systematic review shows clearly that methods are very heterogeneous across studies and suggests that such methodological differences should be considered in a multivariate fashion rather than bivariately. However, subgrouping the included studies in a multivariate manner is unfeasible because it would parcel out studies in a number of cells that would be too small for valid statistical analysis.

Subgroup analyses were not performed on the few studies that evaluated the effect of ICD shock on PTSD development or PTSD symptoms, psychiatric disorders, ICD acceptance and ICD concerns. With the exception of the five studies that assessed PTSD and whose results are mixed as well, the evidences pertaining to the psychiatric diagnosis of mental disorders (4 studies) and to ICD concerns (3 studies) are consistently significant and support the hypothesis that one or more ICD shocks may cause the development of a psychiatric disorder and the hypothesis that shocked patients have significantly more concerns about the ICD. However, the strength of the former evidences is low because the very few studies that tested the effect of ICD shock on mental disorders used a cross-sectional approach. In none of them patients were actually administered the psychiatric interview before ICD implantation and the mental disorders that were diagnosed long after implantation might have been already present before or immediately after, even before the occurrence of the first shock. Despite the severe limitations of a vote-counting approach, the attempt to explore whether methodological differences across studies account for the mixed findings of the literature on the effect of ICD shocks on patients' QoL, anxiety and depression was not vain because it allowed the full discovery of the wide and multiple heterogeneities that exist across studies. Furthermore, it allowed the discovery of severe methodological flaws, the most important of which are undoubtedly the cross-sectional design that was applied by the great part of studies and the multiple wrong ways that were used to operationalize shocks.

Our description is not comprehensive inasmuch as other hypothetical accounting factors were intentionally overlooked. Some information on demographics (age and sex), ICD indication (primary or secondary prevention) and both inclusion and exclusion criteria was extracted from studies and tabulated (Table 1), but any explorative attempt to meta-correlate them with the significance of the shock effect failed. However, in many of the studies that were included in this review, a variety of patient characteristics (demographic, clinical, psychological, etc.) was considered for explaining why, in some patients, QoL and psychological health deteriorate after ICD implantation. Such variables were also entered in multivariate analyses together with ICD shocks, but their effects on patient-centered outcomes were mainly examined as competitors of ICD shocks. Surprisingly, only one study tested the moderating effect of a patient characteristic (i.e., Type-D personality) on the relationship between shocks and psychological distress (anxiety and depression) (Pedersen et al., 2004). A significant interaction (Type-D × Shocks ≥1) was found only for depression, i.e., ICD patients who received one or more shocks and had a type D personality (negative affectivity and social inhibition) reported an higher mean level of depression than ICD patients who received one or more shocks and had not a type D personality. However, this interesting result received no consideration in the discussion, probably because the authors were more concerned in looking beyond shocks toward other determinants such as the type-D personality.

## Conclusions

Clinical practice suggests that ICD shocks have a detrimental effect on patients' QoL and account for the development of anxiety and depression disorders. However, results of studies that have investigated this issue are discordant. The heterogeneity of designs and methods has been ascribed as the main reason for the discrepancy but our findings do not support such hypothesis.

The attempt to solve the problem with a quasi-quantitative approach was daring due to its severe limitations but no other meta-analytic approach was feasible. Regardless of this, the systematic review allowed us to look more clearly at studies and to paint a partial picture of the current status of research on the impact of ICD shocks on patient-centered outcomes.

We think that drawing firm statements about the short, mid and long-term impact of ICD shocks on patients' QoL and psychological well-being is an important matter both for the optimal clinical management of patients and for the adoption of new ICD programming strategies that eliminate or reduce ICD shocks. It is thus imperative that research on the psychological effects of ICD shocks goes further. Future studies should avoid the methodological flaws described in this review and should also consider that the relationship between ICD shocks and patient-centered outcomes may not be as straightforward as expected. Some other putative variables such as personality traits (e.g., Type D personality), coping skills and social support play surely a role and their effects on ICD patients' psychological health should be investigated also in interaction with the occurrence of shocks in order to know the profile of patients who might respond badly and focus treatment resources on them.

### Conflict of interest statement

The authors declare that the research was conducted in the absence of any commercial or financial relationships that could be construed as a potential conflict of interest.
